# Allogeneic hematopoietic stem cell transplantation for VEXAS syndrome: A review and a case report

**DOI:** 10.46989/001c.161719

**Published:** 2026-05-26

**Authors:** Kentaro Nagae, Hiroyuki Muranushi, Yasuhito Nannya, Takeshi Maeda

**Affiliations:** 1 Department of Hematology/Oncology, Kurashiki Central Hospital, Kurashiki, Okayama, Japan; 2 Division of Hematopoietic Disease Control, The Institute of Medical Science, The University of Tokyo, Tokyo, Japan

**Keywords:** VEXAS syndrome, allogeneic hematopoietic stem cell transplantation, EZH2 gene mutation, UBA1 mutation, Literature review

## Abstract

**Background:**

VEXAS (vacuoles, E1 enzyme, X-linked, autoinflammatory, somatic) syndrome is caused by somatic *UBA1* mutations and frequently complicated by myelodysplastic syndrome (MDS). Conventional immunosuppressive and anti-inflammatory therapies are often ineffective, and allogeneic hematopoietic stem cell transplantation (allo-HSCT) is currently the only curative treatment, although its optimal timing and donor selection remain uncertain.

**Case Presentation:**

A 67-year-old man with VEXAS syndrome and MDS harboring *UBA1* (p.M41T) and *EZH2* mutations, refractory to azacitidine and corticosteroids, successfully underwent allo-HSCT from a one-antigen–mismatched related donor. Post-transplant chronic graft-versus-host disease was well controlled with ruxolitinib.

**Methods and Results:**

We reviewed eight studies comprising 45 allo-HSCT recipients with VEXAS syndrome. The median age at transplantation was 59 years, and patients had received a median of five prior systemic therapies. MDS was present in 53.3% of cases, while 42.2% underwent transplantation for VEXAS without overt hematologic malignancy. The most frequent *UBA1* variant was p.Met41Val (37.8%). At last follow-up, 84.4% of patients were alive, with resolution of inflammatory manifestations in most cases.

**Conclusion:**

Allo-HSCT represents a curative option for refractory VEXAS syndrome. Comprehensive genetic profiling may aid in identifying candidates for early transplantation. If early transplantation is required, human leukocyte antigen-mismatched donors may be selected.

## 1. Introduction

VEXAS (vacuoles, E1 enzyme, X-linked, autoinflammatory, somatic) syndrome is caused by a somatic *UBA1* mutation and often associated with myelodysplastic syndrome (MDS).^1–4^ It is a complex systemic disorder involving multiple hematopoietic stem cell clones, which may affect disease progression and treatment response.^5^ The efficacy of conventional immunosuppressive and anti-inflammatory therapies is limited, and allogeneic hematopoietic stem cell transplantation (allo-HSCT) is considered the only curative option.^6,7^ However, evaluation for co-mutations, optimal timing and donor source of HSCT, and post-transplant management remain unclear. To contextualize these unresolved questions, we first present a representative case. This case illustrates the challenges of refractoriness to medical treatment, complexity of clonality, and the difficulty of donor selection, which form the framework for our systematic review.

## 2. Case Presentation

A 67-year-old man visited his primary care physician complaining of recurrent pharyngalgia, conjunctival injection, arthralgia, and fever. Based on the finding of macrocytic anemia with a hemoglobin level of 8.0 g/dL, a hematological disorder was suspected, and the patient was referred to our department for specialized treatment.

**Figure 1.**
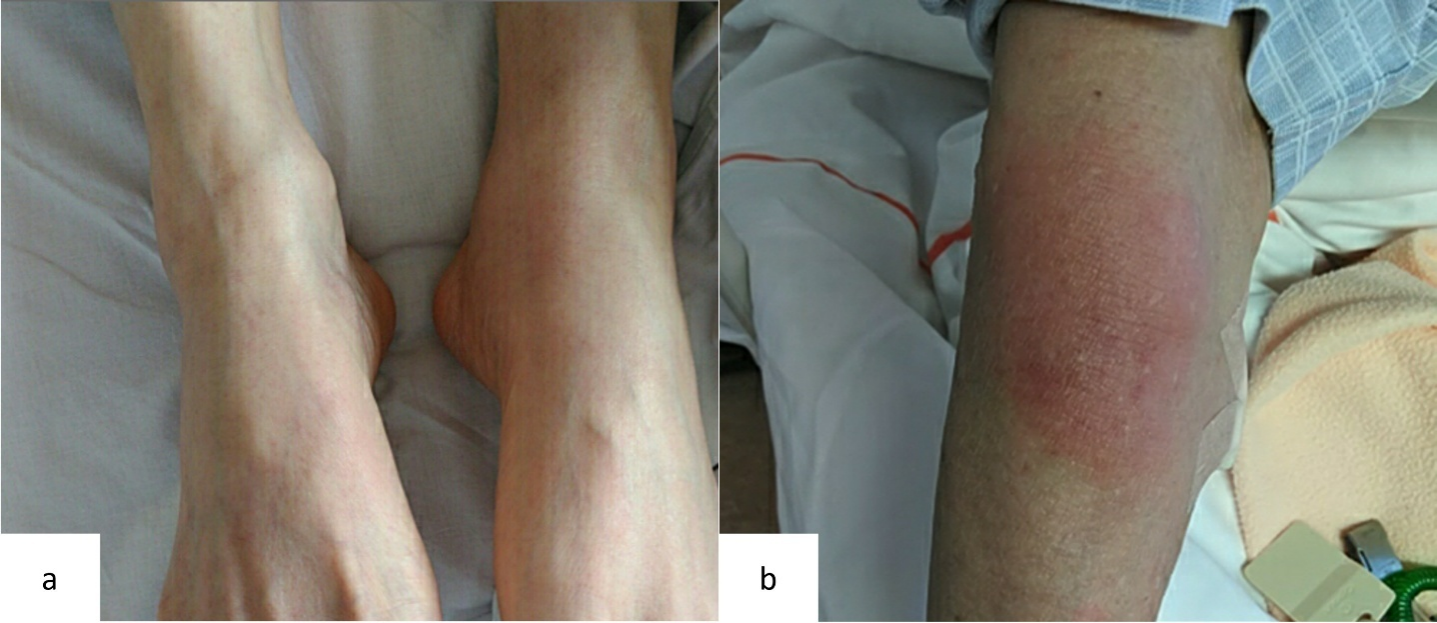
Erythema exhibiting on the joints. The ankles at the time of referral to our department, (b) An elbow after prednisolone was tapered from 25 mg to 10 mg

**Figure 2.**
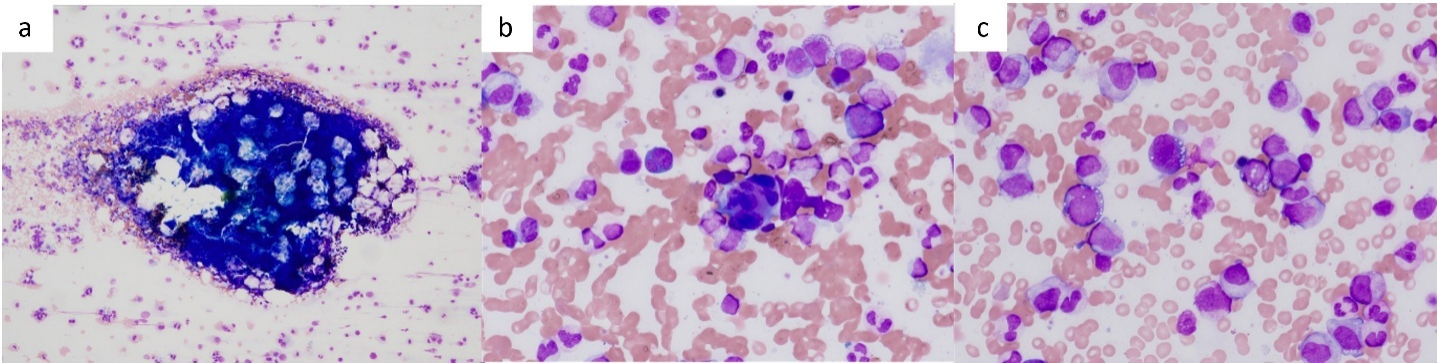
Bone marrow aspiration findings. Hyperplastic bone marrow, May-Giemsa stain 40×magnification, (b) Megakaryocyte dysplasia, May-Giemsa stain 400×magnification, (c) Vacuoles in blood cells, May-Giemsa stain 400×magnification

**Figure 3.**
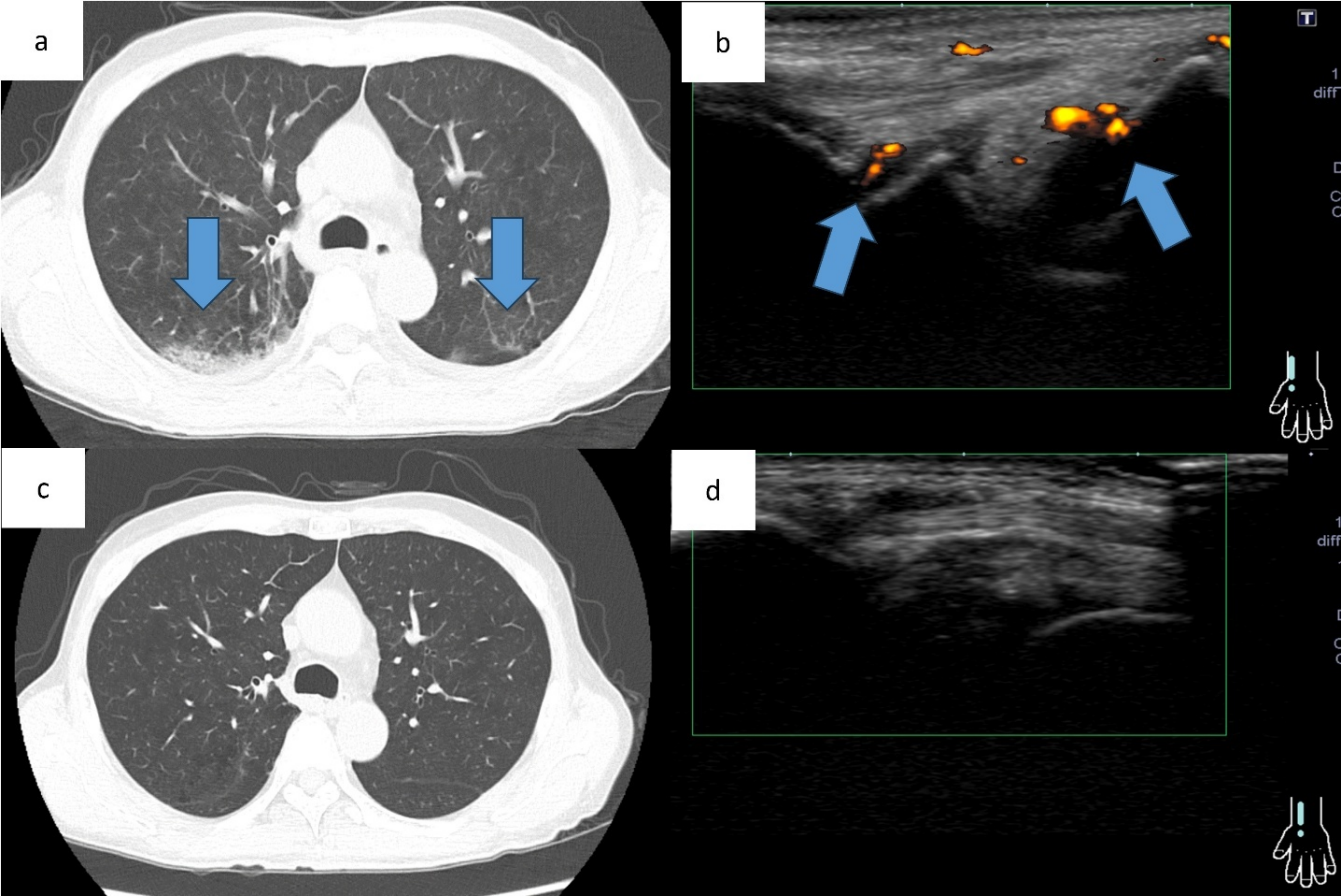
Chest computed tomography and joint ultrasonography images before and after transplantation. Chest computed tomography image before transplantation showing numerous frosted shadows in the periphery of both lungs, (b) Joint ultrasonography image before transplantation showing increased blood flow in the synovial area and arthritis, (c) Chest computed tomography image after transplantation showing the disappearance of the numerous frosted shadows in the periphery of both lungs, (d) Joint ultrasonography image after transplantation showing the disappearance of the arthritis

At the initial visit to our department, he complained of fever and pain in both knees. Erythema was observed on both ankles (Figure 1a), along with redness of the left auricle. Blood tests revealed elevated inflammatory markers, including an increased C-reactive protein level and macrocytic anemia. All tested autoantibodies, such as antinuclear antibodies, antineutrophil cytoplasmic antibodies, and rheumatoid factors, were negative (Table 1). Bone marrow aspiration showed hypercellularity with a nucleated cell count of 251,000/μL, trilineage dysplasia predominantly in the megakaryocytic lineage, and numerous hematopoietic progenitor cells with cytoplasmic vacuoles (Figure 2). Chest computed tomography demonstrated bilateral peripheral ground-glass opacities (Figure 3a). Joint ultrasonography revealed synovial thickening and increased blood flow in the joints (Figure 3b).

**Table 1.** Laboratory results at the time of referral to our department.

TP	6.7 g/dL	RBC	2.80×10^6^ /μL	Haptoglobin	445.2 mg/dL	Protein fraction	
Alb	2.6 g/dL	MCV	104.3 fL	Zn	48 μg/dL	Alb	47.50%
T-Bil	0.4 mg/dL	Ht	29.20%	Cu	160 μg/dL	α1	6.80%
AST	18 U/L	Hb	9.3 g/dL	VitB12	2560 pg/mL	α2	14.10%
ALT	23 U/L	Reti	1.60%	Erythropoietin	105 mIU/mL	β1	10.40%
LDH	153 U/L	WBC	8.6×10^3^ /μL	Ferritin	447 mg/dL	γ	21.20%
ALP	153 U/L	PLT	29.9×10^4^ /μL	FE	28 μg/dL	A/G	0.63
γ-GT	31 U/L	Eos	0%	UIBC	111 μg/dL		
UA	4.1 mg/dL	Lymph	14.4%	CRP	11.27 mg/dL		
Cr	0.7 mg/dL	Blast	0%	WT1	92 copy/μgRNA		
BUN	13 mg/dL	Neutro	7224 /μL	IgG	1268 mg/dL	Bone marrow	
Na	137 mmol/L			IgG4	23 mg/dL	NCC	25.1×10^4^ /μL
K	4.3 mmol/L			IgA	276 mg/dL	MgK	110 /μL
				IgM	108 mg/dL	M/E ratio	9.7
				C3	125.6 mg/dL	Myeloblast	0.20%
				C4	36.8 mg/dL	Ringed sideroblasts	1%
				RF	4 IU/mL		
				MMP-3	68.8 ng/mL		
				KL-6	227 U/mL		
				ANCA	Negative		

**Abbreviations:** TP, total protein; Alb, albumin; T-Bil, total bilirubin; AST, aspartate aminotransferase; ALT, alanine aminotransferase; LDH, lactate dehydrogenase; ALP, alkaline phosphatase; γ-GT, gamma-glutamyl transferase; BUN, blood urea nitrogen; Cr, creatinine; RBC, red blood cells; WBC, white blood cells; Hb, hemoglobin; Ht, hematocrit; MCV, mean corpuscular volume; PLT, platelets; Reti, reticulocytes; FE, serum iron; UIBC, unsaturated iron-binding capacity; CRP, C-reactive protein; WT1, Wilms tumor 1; NCC, nucleated cell count; MgK, megakaryocytes; M/E ratio, myeloid-to-erythroid ratio; ANCA, anti-neutrophil cytoplasmic antibody.

**Figure 4.**
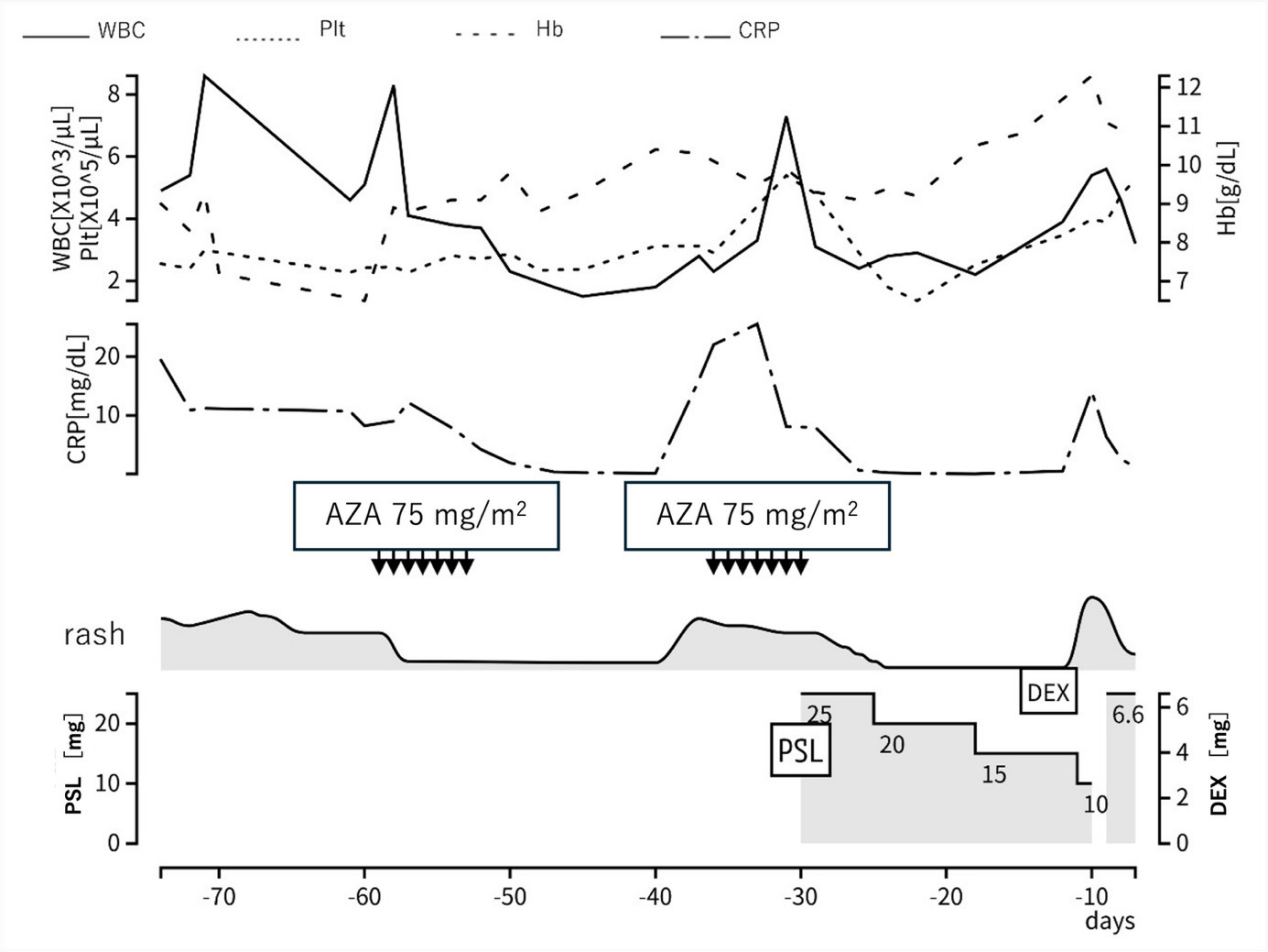
Clinical course before transplantation. (day 0 = day of transfusion) **Abbreviations:** WBC, white blood cell; Plt, platelet; Hb, hemoglobin; CRP, C-reactive protein; AZA, azacitidine; PSL, prednisolone; DEX, dexamethasone

Based on these findings, a provisional diagnosis of MDS complicated by an autoimmune disorder was made. Azacitidine was started to control the disease. The initial administration of azacitidine temporarily improved the erythema and inflammatory findings (Figure 4). However, symptoms recurred, so the treatment interval was shortened to 5 days for the second course. The second course was less effective than the first course. Then, prednisolone 0.5 mg/kg was started. His symptoms improved, and the dose of prednisolone was gradually tapered. However, symptoms recurred when the dose was tapered to 10 mg/day (Figure 1b). Therefore, dexamethasone 6.6 mg was added.

**Figure 5.**
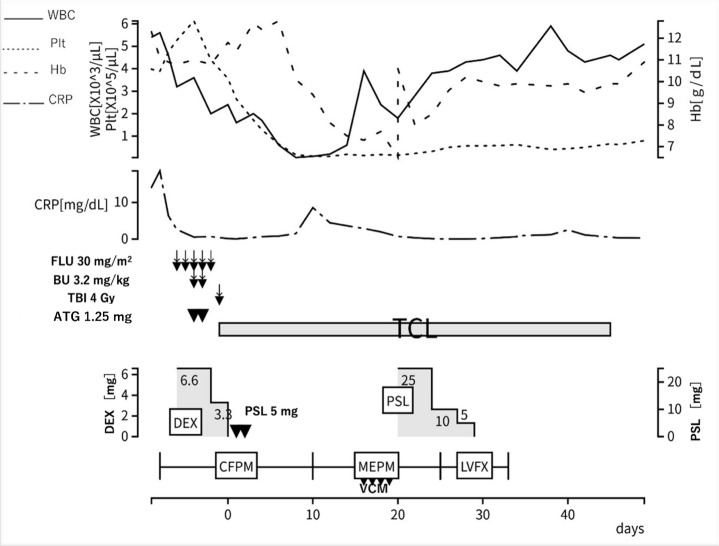
Clinical course after transplantation. (day 0 = day of transfusion) **Abbreviations:** WBC, white blood cell; Plt, platelet; Hb, hemoglobin; CRP, C-reactive protein; FLU, fludarabine; BU, busulfan; TBI, total body irradiation; ATG, anti-thymocyte globulin; TCL, tacrolimus; DEX, dexamethasone; PSL, prednisolone; CFPM, cefepime; MEPM, meropenem; VCM, vancomycin; LVFX, levofloxacin

At this point, comprehensive gene analysis using next generation sequencing^8^ performed on the bone marrow sample identified somatic mutations in the *UBA1* p.M41T (variant allele frequency 0.64) and *EZH2* p.N676fs (variant allele frequency 0.16) genes. Based on these findings, a diagnosis of VEXAS syndrome associated with MDS was made. Considering the resistance to azacitidine and prednisolone, allo-HSCT was planned as a curative treatment (Figure 5). The conditioning regimen consisted of fludarabine (150 mg/m²), busulfan (6.4 mg/kg), and total body irradiation (4 Gy). The patient underwent an allo-HSCT from his one human leukocyte antigen (HLA)-mismatched daughter. Tacrolimus, short-term methotrexate, and antithymocyte globulin (ATG) (1.25 mg/kg for 2 days) were administered for graft-versus host disease (GVHD) prophylaxis. The Hematopoietic Cell Transplantation-Comorbidity Index score was 4 before transplantation due to decreased respiratory and hepatic function.

Engraftment of hematopoietic stem cells was confirmed on Day 14. Engraftment syndrome developed on Day 18, but rapidly improved with the administration of prednisolone 0.5 mg/kg (25 mg). The patient’s general condition was stable, and he was discharged on Day 50. On Day 81, however, erythema appeared on his face and scalp and spread to his limbs and trunk. He was diagnosed with acute skin GVHD (stage 3), and prednisolone 0.5 mg/kg (25 mg) was restarted on Day 95, resulting in rapid improvement of the erythema. When prednisolone was tapered to 7 mg, lichen planus-like skin lesions appeared, and he was diagnosed with chronic GVHD (NIH skin score 2). Prednisolone was increased again to 15 mg on Day 172, but the skin lesions showed poor improvement. Liver dysfunction (AST 146 U/L, ALT 202 U/L, γ-GTP 1082 U/L, NIH score 1) also developed. Ruxolitinib 20 mg was introduced as the second-line treatment on Day 180, resulting in improvement of both the skin lesions and liver dysfunction. Subsequently, prednisolone was tapered to 6 mg, and there has been no GVHD recurrence.

At post-transplant follow-up, the peripheral ground-glass opacities on chest computed tomography and arthritis on joint ultrasonography observed before transplantation had resolved (Figure 3cd). Hematological counts and inflammatory markers have remained stable. To date, there have been no clinical or radiological findings suggestive of a recurrence of VEXAS syndrome.

## 3. Methods of systematic review

A literature search was conducted on PubMed from its inception until August 2025. The keywords “VEXAS” and “allogeneic hematopoietic stem cell transplantation” were used. Eligible studies included retrospective and prospective studies, case reports, and case series of VEXAS syndrome treated with allo-HSCT. Review articles, studies lacking data on treatment, treatment response, and individual patient data were excluded. Eight studies were selected for review, from which data on the clinical characteristics, number of pre-transplant treatment regimens, and transplant information of 45 patients were extracted (Table 2).^9–17^

**Table 2.** Inclusion and exclusion criteria for the systematic review.

Category	Criteria
Database searched	PubMed (from inception to August 2025)
Search keywords	“VEXAS” AND “allogeneic hematopoietic stem cell transplantation”
Study types included	Case reports, case series, and cohort studies reporting allo-HSCT for VEXAS syndrome
Population	Patients diagnosed with VEXAS syndrome confirmed by a *UBA1* mutation
Intervention	Allo-HSCT (any donor source, conditioning regimen, or GVHD prophylaxis)
Outcomes extracted	Survival, resolution of VEXAS symptoms, GVHD incidence, donor type, conditioning regimen
Exclusion criteria	(1) Duplicate data from overlapping institutions or authorship groups, (2) Studies without extractable patient-level data or post-transplant outcomes, and (3) Review-only or descriptive papers without case-level evidence

**Abbreviations:** allo-HSCT, allogeneic hematopoietic stem cell transplantation; GVHD, graft-versus-host disease

## 4. Results of systematic review

### 4-1. Patient Cohort and Disease Characteristics

A total of 45 cases of VEXAS syndrome treated with allo-HSCT from eight studies (Table 3) were analyzed. The median age at transplantation was 59 years (range: 46–70 years). MDS was the most common underlying hematologic disorder, present in 24 cases (53.3%); 19 cases (42.2%) were treated for VEXAS syndrome alone, in the absence of overt MDS/myeloid neoplasms.

**Table 3.** Summary of individual cases undergoing allo-HSCT for VEXAS syndrome.

**Author**	***UBA1* variant**	**Primary disease**	**Age at diagnosis**	**Age at HSCT**	**Donor type**	**Graft origin**	**Conditioning regimen**	**GVHD prophylaxis**	**Outcome**
Diarra^9^	p.Met41Val (c.121A>G)	MDS	43	46	MUD	PB	Flu/Bu	ATG/CsA/MMF	Alive
	p.Met41Val (c.121A>G)	MF	56	59	RD	BM	Flu/Bu	CsA/MTX	Alive
	p.Met41Leu(c.121A>C)	MDS	63	65	MUD	PB	Flu/Bu	CsA/MMF	Alive
	p.Met41Thr (c.122T>C)	MDS	48	50	MUD	PB	Flu/Bu	ATG/CsA/MTX	Alive
	p.Met41Val (c.121A>G)	MDS	56	58	RD	PB	Flu/Bu/TT	PTCy/CsA/MMF	Alive
	p.Met41Val (c.121A>G)	MDS/MF	50	55	MUD	PB	Bu/Cy	ATG/CsA/MTX	Dead
Mangaonkar^10^	p.Met41Thr (c.122T>C)	VEXAS only	NA	63	MUD	NA	Flu/Mel	PTCy/Tac/MMF	Alive
	p.Met41Val (c.121A>G)	VEXAS only	NA	60	MSD	NA	Flu/Mel	PTCy/Tac/MMF	Alive
	C.C118-1g>c	VEXAS only	NA	59	MSD	NA	Flu/Mel	Tac/MTX	Alive
	p.Met41Thr (c.122T>C)	VEXAS only	NA	74	MUD	NA	Flu/Mel	PTCy/Tac/MMF	Alive
	p.Met41Thr (c.122T>C)	MDS	NA	49	MUD	NA	Flu/Mel	PTCy/Tac/MMF	Alive
	p.Met41Val (c.121A>G)	VEXAS only	NA	65	MSD	NA	Flu/Mel	PTCy/Tac/MMF	Alive
	p.Met41Val (c.121A>G)	VEXAS only	NA	63	MUD	NA	Flu/Bu	PTCy/Tac/MMF	Alive
	p.Met41Val (c.121A>G)	VEXAS only	NA	70	MUD	NA	Flu/Mel	PTCy/Tac/MMF	Alive
	p.Met41Val (c.121A>G)	VEXAS only	NA	60	MSD	NA	Flu/Bu	PTCy/Tac/MMF	Alive
	C.C118-1g>c	VEXAS only	NA	69	Haplo	NA	Flu/BU/TT	PTCy/Tac/MMF	Alive
Al-Hakim^11^	p.Met41Val (c.121A>G)	MDS	49	51	Haplo	PB	Flu/BU/TT	PTCy/Tac/MMF	Dead
	p.Met41Val (c.121A>G)	VEXAS only	64	67	MUD	PB	Flu/Mel	CsA/CAM	Alive
	p.Met41Thr (c.122T>C)	VEXAS only	52	61	MSD	PB	Flu/Treo	CsA/MMF/CAM	Dead
van Leeuwen-Kerkhoff^12^	p.Met41Thr (c.122T>C)	VEXAS only	51	52	MMUD	NA	Flu/Mel/TT	MMF/ATG/PSL	Alive
Loschi^13^	p.Met41Leu(c.121A>C)	VEXAS only	69	70	MMUD	PB	Flu/Bu	PTCy/CsA/MMF	Alive
Gurnari^14^	p.Met41Thr (c.122T>C)	MDS	NA	69	MUD	PB	Flu/Bu	ATG/CsA/ MTX	Alive
	c.118-2A>C	MDS	NA	53	MUD	PB	Flu/Bu	ATG/CsA/MTX	Alive
	p.Met41Thr (c.122T>C)	VEXAS only	NA	52	MMUD	PB	Flu/Mel/TT	ATG/MMF+ TCRαβ/CD19 depletion	Alive
	p.Met41Thr (c.122T>C)	VEXAS only	NA	61	MRD	PB	Flu/Treo	CAM/CNI/ MMF	Dead
	p.Met41Leu(c.121A>C)	VEXAS only	NA	52	MUD	PB	Flu/Bu	ATG/CNI/MMF	Alive
	p.Met41Val (c.121A>G)	VEXAS only	NA	52	MMRD	PB	Flu/Bu/TT	PTCy/CNI/MMF	Dead
	p.Met41Val (c.121A>G)	VEXAS only	NA	67	MUD	PB	Flu/Mel	CAM/CsA	Dead
	p.Met41Val (c.121A>G)	MPN	NA	59	MRD	BM	Flu/Mel/TBI	CsA/MTX	Alive
	p.Met41Leu(c.121A>C)	MDS	NA	65	MUD	PB	Flu/Bu	PTCy/CNI/MMF	Alive
	p.Met41Val (c.121A>G)	MDS	NA	46	MUD	PB	Flu/Bu	ATG/CNI/MMF	Alive
	p.Met41Thr (c.122T>C)	MDS	NA	50	MUD	PB	Flu/Bu	ATG/PTCy/ CsA/MTX	Alive
	p.Met41Val (c.121A>G)	MDS	NA	60	MUD	PB	Flu/Treo	PTCy/CNI/MMF	Alive
	NA	MDS	NA	61	MUD	PB	Flu/Bu	ATG/CsA/MMF	Alive
	p.Met41Thr (c.122T>C)	MDS	NA	65	MUD	PB	Flu/Treo	ATG/CsA/MTX	Alive
	p.Met41Thr (c.122T>C)	MDS	NA	55	MUD	PB	Flu/Bu/Cy/Amsa/AraC	ATG/CsA/MTX	Dead
	p.Ser56Phec.167C>T;	MDS	NA	59	MRD	PB	Flu/Bu/Cy/Amsa/AraC	ATG/CNI/MMF	Alive
	p.Met41Val (c.121A>G)	MDS	NA	59	MMRD	PB	Flu/Bu/TT	PTCy/CNI/MMF	Alive
	C.C118-1g>c	MDS	NA	59	MMRD	PB	Flu/Bu/TT	PTCy/CNI/MMF	Alive
	p.Met41Thr (c.122T>C)	MDS	NA	59	MUD	PB	Flu/Treo	ATG/CsA/MTX	Alive
Stiburkova^15^	c.1430G>C in exon 14 (p.Gly477Ala)	MDS	NA	59	MUD	PB	Flu/Treo	ATG/CsA/MMF	Alive
Gurnari^16^	p.Met41Thr (c.122T>C)	MDS	58	NA	MUD	NA	Flu/Treo	NA	Alive
	p.Met41Thr (c.122T>C)	MDS	65	NA	MUD	NA	Flu/Treo	NA	Alive
	p.Met41Val (c.121A>G)	MDS	59	NA	MUD	NA	Flu/Treo	NA	Alive
Bellman^17^	Met41Thr (c.122 T>C)	MDS	66	NA	Haplo	PB	Flu/TBI	PTCy/Tac/MMF	Alive

**Abbreviations:** allo-HSCT, allogeneic hematopoietic stem cell transplantation; MDS, myelodysplastic syndrome; MF, myelofibrosis; MPN, myeloproliferative neoplasm; MUD, matched unrelated donor; MSD, matched sibling donor; MMUD, mismatched unrelated donor; MRD, matched related donor; MMRD, mismatched related donor; Haplo, haploidentical donor; PB, peripheral blood; BM, bone marrow; Flu, fludarabine; Bu, busulfan; Cy, cyclophosphamide; Mel, melphalan; Treo, treosulfan; TT, thiotepa; TBI, total body irradiation; Amsa, amsacrine; AraC, cytarabine; ATG, anti-thymocyte globulin; CsA, cyclosporine A; Tac, tacrolimus; MMF, mycophenolate mofetil; MTX, methotrexate; PTCy, post-transplant cyclophosphamide; CAM, campath (alemtuzumab); CNI, calcineurin inhibitor; PSL, prednisolone; NA, not available.

The p.Met41Val variant was the most common variant, occurring in 17 cases (37.8%), followed by the p.Met41Thr variant in 14 cases (31.1%) and the p.Met41Leu variant in 4 cases (8.9%).

### 4-2. Transplant Characteristics and Pre-transplant Treatment

A median of five (range: 0–13) systemic drug regimens consisting primarily of immunosuppressants were administered before transplantation. Reduced-intensity conditioning was the predominant approach in most cases. For graft-versus-host disease (GVHD) prophylaxis, post-transplant cyclophosphamide was administered in 20 cases (44.4%). Most transplants were from HLA-matched donors, but three were from haploidentical donors. Most patients received peripheral blood stem cell transplant, but two received bone marrow transplant.

### 4-3. Post-transplant Outcomes

At the time of reporting, 38 (84.4%) of the 45 cases were alive, in most of whom surviving clinical symptoms resolved. Ruxolitinib and belumosudil were administered for post-transplant complications, including acute and chronic GVHD, and recurrent inflammation.

## 5. Integrated Discussion and Treatment Strategy

### 5-1. Limitations of Drug Therapy and Need for Transplantation

Patients with VEXAS syndrome typically receive multiple immunosuppressants before definitive diagnosis is established.^1^ Although these treatments may alleviate symptoms and temporarily improve quality of life, they do not eradicate the underlying *UBA1*-mutant hematopoietic clone, inherently limiting their ability to achieve sustained disease control. Consistent with this limitation, our review showed that many patients had received multiple systemic therapies prior to transplantation, highlighting the refractory nature of VEXAS syndrome to medical treatment.

In the present case, azacitidine and corticosteroids resulted in only transient clinical improvement, followed by early steroid dependence. These findings suggest that long-term disease control with drug therapy alone would be difficult. Supporting this perspective, a recent meta-analysis has shown that allo-HSCT represents a reliable therapeutic option for selected patients with VEXAS syndrome.^18^

### 5-2. Transition from Drug Therapy to Transplantation

Determining the optimal timing for transition from drug therapy to allo-HSCT is one of the most challenging aspects of managing VEXAS syndrome. Initial treatment strategies typically focus on disease control using agents such as corticosteroids and azacitidine,^4,5,19^ but long-term disease control is often elusive, and many patients eventually develop treatment refractoriness or steroid dependence.^4^ Once resistance to drug therapy or steroid dependence becomes evident, allo-HSCT is regarded as the only curative option. At this stage, the therapeutic goal shifts from achieving prolonged disease control to preserving performance status and minimizing treatment-related toxicity in preparation for allo-HSCT.^6^

In the present case, early recognition of azacitidine resistance and steroid dependence prompted a timely transition to a transplant-oriented strategy. Consequently, allo-HSCT was performed while the patient maintained good performance status, despite his advanced age.

### 5-3. Importance of Personalized Treatment Based on Genetic Mutation Profiles

Emerging evidence suggests that the clinical phenotype and prognosis of VEXAS syndrome vary according to the type of *UBA1* mutation.^3^ In our review, the p.Met41Val variant, which was associated with poor prognosis, was the most frequently observed mutation among transplanted cases. In addition to *UBA1* mutations, VEXAS syndrome is frequently accompanied by clonal hematopoiesis involving additional CHIP-associated gene mutations. Gutierrez-Rodrigues et al. reported a high prevalence of mutations in genes such as *DNMT3A*, *TET2*, and *ASXL1*, underscoring the genetic complexity of this disorder.^20^

In the present case, an *EZH2* mutation was identified in addition to the *UBA1* mutation. This co-mutation provides a plausible molecular explanation for the observed resistance to azacitidine.^21,22^ These findings emphasize the importance of viewing VEXAS syndrome not as a disease driven solely by a single *UBA1* mutation, but rather as a disorder arising from complex, polyclonal hematopoiesis. This understanding is crucial when formulating individualized treatment strategies, particularly with respect to assessing the limitations of drug therapy and determining the timing of allo-HSCT.

### 5-4. Donor Source

In allo-HSCT, an HLA-matched donor is generally considered optimal. In our review, most patients underwent transplantation from HLA-matched donors. However, because VEXAS syndrome predominantly affects the elderly, the availability of HLA-matched sibling donors is often limited. In the present case, allo-HSCT from his one-antigen HLA-mismatched daughter resulted in successful engraftment and durable remission. This suggests that transplantation may remain a feasible option even in patients with limited donor availability.

### 5-5. Conditioning Regimen

Patients with VEXAS syndrome are typically of advanced age and frequently have comorbidities. As a result, reduced-intensity conditioning (RIC) regimens are commonly favored. Accordingly, most cases included in our review received RIC regimens. In the present case, a RIC regimen consisting of fludarabine, low dose busulfan, and reduced total body irradiation was selected based on his age and comorbidities. At present, an optimal conditioning regimen specific to VEXAS syndrome has not been established, and further accumulation of clinical experience is required.

### 5-6. GVHD Prophylaxis

The primary objective of allo-HSCT for VEXAS syndrome is to replace the *UBA1*-mutant clone with normal donor-derived stem cells. Unlike acute myeloid leukemia, a strong graft-versus-tumor effect is not necessarily required; therefore, stable engraftment with minimal toxicity represents the highest priority.

At the time this case was treated, post-transplant cyclophosphamide (PTCy) was approved in Japan only for HLA-haploidentical transplantation, and ATG was therefore used for GVHD prophylaxis. In recent years, however, PTCy has been widely adopted for GVHD prophylaxis across various transplant settings, including one-allele HLA-mismatched and HLA-matched transplantation.^23,24^ Emerging data suggest that PTCy may also be useful in selected patients with VEXAS syndrome.

### 5-7. Post-transplant Treatment

GVHD remains a major post-transplant complication in patients with VEXAS syndrome. A recent meta-analysis reported an incidence of grade II–IV acute GVHD of 42% and chronic GVHD of 13%, underscoring the importance of effective post-transplant management.^17^ Ruxolitinib, a Janus kinase (JAK) inhibitor, has emerged as a promising therapeutic option in this setting.^25,26^ It is not only effective for steroid-refractory GVHD, but also for the inflammatory state of VEXAS syndrome.^27,28^ In the present case, use of ruxolitinib resulted in improvement of cutaneous and hepatic GVHD, without recurrence of VEXAS-related inflammatory symptoms. Although evidence remains limited, post-transplant ruxolitinib may contribute to improved outcomes.

### 5-8. What the Present Case Adds Beyond the Review

In our review of 45 patients with VEXAS syndrome who underwent allo-HSCT, the median age at transplantation was 59 years, and patients had received a median of five systemic treatment regimens prior to transplantation. Most transplants were performed using HLA-matched donors, and PTCy was used for GVHD prophylaxis in some patients.

In contrast, the present patient underwent transplantation at 67 years of age (older than the cohort median) after only two lines of pre-transplant therapy, while maintaining good performance status. A one-antigen mismatched related donor was selected, and favorable outcomes were achieved using ATG-based GVHD prophylaxis and post-transplant ruxolitinib.

This contrast suggests that early, genetics-informed decision-making based on treatment response may improve transplant outcomes even in older patients. Notably, the presence of an *EZH2* mutation in this case provided a molecular rationale for resistance to drug therapy and supported the decision to proceed to allo-HSCT. This case therefore adds meaningful clinical insight to our review by illustrating individualized decision-making processes that are not readily captured by aggregated data alone.

## 6. Conclusion

In summary, allo-HSCT should be considered when drug therapy is ineffective or intolerable. Genetic mutation profiles may serve as a decision-making tool. When an optimal HLA-matched donor is not available, an HLA-mismatched donor may be acceptable. Use of ATG or PTCy may enable safer allo-HSCT. Ruxolitinib may be effective for both controlling GVHD and managing VEXAS-related inflammatory symptoms. Further accumulation of cases is necessary to establish a treatment strategy for VEXAS syndrome.

### Author Contribution – per CRediT

Methodology: Kentaro Nagae (Equal), Hiroyuki Muranushi (Equal). Formal Analysis: Kentaro Nagae (Equal), Hiroyuki Muranushi (Equal). Investigation: Kentaro Nagae (Equal), Hiroyuki Muranushi (Equal). Writing – original draft: Kentaro Nagae (Lead). Resources: Kentaro Nagae (Equal), Hiroyuki Muranushi (Equal), Yasuhito Nannya (Equal). Conceptualization: Hiroyuki Muranushi (Lead). Writing – review & editing: Hiroyuki Muranushi (Equal), Yasuhito Nannya (Equal), Takeshi Maeda (Equal). Supervision: Takeshi Maeda (Lead).

### Competing of Interest – COPE

The authors have no relevant financial or non-financial interests to disclose.

### Ethical Conduct Approval – Helsinki – IACUC

This case report was created in line with the principles of the Declaration of Helsinki. Approval was granted by the Ethics Committee of Kurashiki Central Hospital (No. 4703).

### Consent to participate / Consent to publish

The participant has consented to the submission of the case report to the journal.
